# *Fusobacterium nucleatum* Accelerates the Progression of Colitis-Associated Colorectal Cancer by Promoting EMT

**DOI:** 10.3390/cancers12102728

**Published:** 2020-09-23

**Authors:** Mi Ra Yu, Hye Jung Kim, Hae Ryoun Park

**Affiliations:** 1 Department of Oral Pathology, BK21 PLUS Project, School of Dentistry, Pusan National University, Yangsan 50612, Korea; mira0802@pusan.ac.kr; 2 Periodontal Disease Signaling Network Research Center (MRC), School of Dentistry, Pusan National University, Yangsan 50612, Korea

**Keywords:** colorectal cancer, EMT, *F. nucleatum*, EGFR, DSS, AOM/DSS

## Abstract

**Simple Summary:**

Colitis-associated cancer (CAC) are associated with the development and progression of colorectal cancer (CRC). And *Fusobacterium nucleatum* (*F. nucleatum*), a major pathogen involved in chronic periodontitis, may play an important role in CRC progression. Though the importance of *F. nucleatum* in CRC has attracted attention, its exact role and related mechanism in CAC progression remain unclear. We investigated the effects of *F. nucleatum* in both in vitro and in vivo colitis models induced with dextran sodium sulfate (DSS), a well-known colitis-inducing chemical, on the aggressiveness of CAC and its related mechanism. This study showed that *F. nucleatum* accelerates the progression of CAC cancer by promoting epithelial–mesenchymal transition (EMT). This study provides a novel mechanism involved *F. nucleatum* in the development of colitis-associated CRC.

**Abstract:**

Recently, it has been reported that *Fusobacterium nucleatum*, a major pathogen involved in chronic periodontitis, may play an important role in colorectal cancer (CRC) progression. In addition, inflammatory bowel diseases such as ulcerative colitis and Crohn’s disease represent major predisposing conditions for the development of CRC, and this subtype of cancer is called colitis-associated cancer (CAC). Although the importance of *F. nucleatum* in CRC has attracted attention, its exact role and related mechanism in CAC progression remain unclear. In this study, we investigated the effects of *F. nucleatum* in experimental colitis induced with dextran sodium sulfate (DSS), which is a well-known colitis-inducing chemical, on the aggressiveness of CAC and its related mechanism in both in vitro and in vivo models. *F. nucleatum* synergistically increased the aggressiveness and epithelial–mesenchymal transition (EMT) characteristics of CRC cells that were treated with DSS compared to those in non-treated CRC cells. The role of *F. nucleatum* in CAC progression was further confirmed in mouse models, as *F. nucleatum* was found to significantly increase the malignancy of azoxymethane (AOM)/DSS-induced colon cancer. This promoting effect of *F. nucleatum* was based on activation of the EGFR signaling pathways, including protein kinase B (AKT) and extracellular signal-regulated kinase (ERK), and epidermal growth factor receptor (EGFR) inhibition significantly reduced the *F. nucleatum*-induced EMT alteration. In conclusion, *F. nucleatum* accelerates the progression of CAC by promoting EMT through the EGFR signaling pathway.

## 1. Introduction

Colorectal cancer (CRC) is a fatal malignancy that is frequently diagnosed worldwide [1]. Both hereditary and environmental risk factors play a part in the development of CRC. Although genetic mutations have recently been found to play a key role in colon carcinogenesis, environmental factors also contribute to the development and progression of CRC [2]. In a large number of experimental, clinical, and epidemiologic studies, the incidence of non-familial CRC has been inextricably associated with dietary and lifestyle factors, such as red meat consumption, the use of artificial preservatives, and smoking [3].

Chronic inflammation has been found to play a key role in the progression of colon cancers. Numerous studies have shown that inflammatory bowel diseases (IBDs), including Crohn’s disease and ulcerative colitis (UC), are associated with the development and progression of CRC [4,5,6]. It has been reported that 25% of patients with IBD have colon cancer [7,8]. Based on the evidence of a strong association between IBD and CRC, colitis-associated cancer (CAC) has been proposed and is currently recognized as a special subtype of CRC.

Parallelly, there has been an interest in defining gut microbiota that are linked to colon cancer [9]. With advances in sequencing techniques, studies have reported correlations between microbiota dysbiosis and CRC, and pathogenic microbiota that accelerate colon cancer progression have been identified [10]. For example, it has been reported that the enterotoxigenic *Bacteroides fragilis* contribute to colon carcinogenesis by producing toxins [11]. Together with numerous potential carcinogenic intestinal microorganisms, *Fusobacterium nucleatum* (*F. nucleatum*) has been reported to be a contributing factor to CRC progression based on its presence in CRC specimens [12,13,14]. The importance of *F. nucleatum* in CRC is further supported by reports that it increases the chemoresistance of colon cancer via modulating autophagy and enhances the proliferation and tumor development of CRC by activating Toll-like receptor 4 (TLR4) signaling [9]. In addition, studies have demonstrated a relationship between *F. nucleatum* and colitis by showing that *F. nucleatum* aggravates colitis via damaging epithelial integrity and regulating M1 macrophage polarization [15]. However, despite such a focus on clarifying the role of *F. nucleatum* in the development and progression of colon diseases, including colitis and CRC, there are few studies exploring the correlation between them, and the mechanism involved remains unclear. Since *F. nucleatum* has been shown to modulate the progression of both CRC and colitis, it has been hypothesized that *F. nucleatum* may drive the progression of UC and the development of CAC. Here, we investigated the contribution of *F. nucleatum* to CAC progression by assessing the phenotypic changes in CRC cells treated with dextran sulfate sodium (DSS), a colitis-inducing chemical, in the presence of *F. nucleatum*. In addition, the effect of *F. nucleatum* on azoxymethane (AOM)/DSS-induced ulcerative colitis colorectal cancer (UC-CRC) in a C57BL/6 mouse model was examined.

## 2. Results

### 2.1. F. nucleatum Promotes Growth in CRC Cells under DSS Stimulation

**Figure 1:**
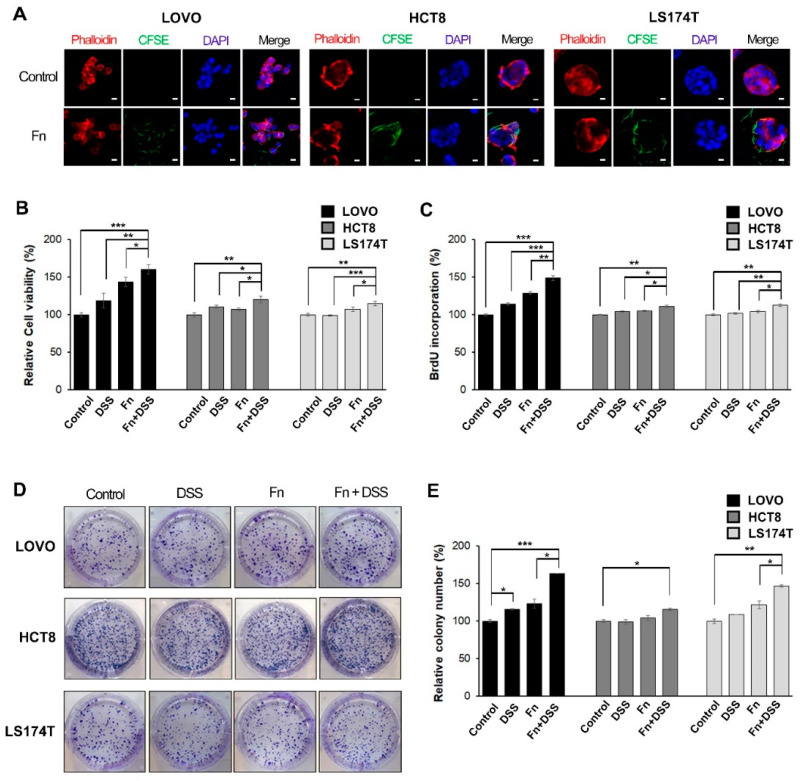
*F. nucleatum* promotes growth in colorectal cancer (CRC) cells under DSS stimulation. (**A**) LOVO, HCT8, and LS174T colorectal cancer cells (CRCs) were infected with carboxyfluorescein succinimidyl ester (CFSE)-labeled *F. nucleatum* (green) for 3 h before analysis using confocal microscopy. The plasma membrane (rhodamine-phalloidin) and nucleus (DAPI) were stained red and blue, respectively. CFSE-labeled *F. nucleatum* was found mainly in the cytosol or the boundary of the cell; 200 times magnification; scale bar: 10 μm. (**B**) CRC cells were incubated with the vehicle, DSS, *F. nucleatum*, or DSS + *F. nucleatum*. Cell-proliferation rates were evaluated using MTT assays. (**C**) Proliferation was assessed using a BrdU incorporation assay. Relative changes in the percentage of the absorbance at 450 nm compared to that in the uninfected control. (**D**) Images showing the effect of *F. nucleatum* on the colony-forming ability of CRC cells. (**E**) Graphical representation of the number of colonies formed expressed as percentage of the control. The graph represents relative levels from three independent experiments. The data are presented as the mean ± SEM (* *p* < 0.05, ** *p* < 0.01, and *** *p* < 0.001; unpaired Student’s *t*-test). Abbreviations: DSS, dextran sodium sulfate; Fn, *F. nucleatum*.

To evaluate the ability of *F. nucleatum* to invade CRC cells, LOVO, HCT8, and LS174T cells were co-cultured with CFSE-stained *F. nucleatum* for 3 h, and then, the cells were washed with fresh media containing antibiotics. The optimal dose of *F. nucleatum* for invasion into CRC cells was determined based on the expression of *F. nucleatum* 16S rRNA. There was a significant increase in the invasion of CRC cells by *F. nucleatum* at a multiplicity of infection (MOI) of 500 compared with that at a MOI of 125 or 250 (Figure S1A). The internalization of *F. nucleatum* in CRC cells was confirmed by conforcal orthogonal view (Figure S1B). The cells were fixed and visualized using confocal microscopy. The internalization of *F. nucleatum* into CRC cells after infection was mainly observed within the cytosol or the boundary of the cell (Figure 1A).

We hypothesized that *F. nucleatum* affects CAC progression by stimulating the proliferative activity of CRC cells that have been previously exposed to inflammatory conditions. To test this hypothesis, *F. nucleatum*-infected CRC cells were treated with DSS, a well-known colitis-inducing agent, for 24 h; then, the viability of CRC cells was assessed using 3-(4,5-dimethylthiazol-2-yl)-2,5-diphenyltetrazolium bromide (MTT assays). Cell viability was significantly higher in *F. nucleatum*-infected CRC cells treated with DSS than in CRC cells treated with DSS or *F. nucleatum* alone (Figure 1B). Treatment with *F. nucleatum* alone increased cell viability in LOVO cells, but there was no change in HCT8 and LS174T cells (Figure 1B). To confirm that *F. nucleatum* promotes the growth of CRC cells, we performed bromodeoxyuridine (BrdU) incorporation assays and colony-forming assays. As shown in Figure 1C, *F. nucleatum* significantly increased BrdU incorporation in CRC cells under DSS treatment, especially in LOVO cells. The percentage of colony-forming cells was determined for each experimental condition. *F. nucleatum*-infected CRC cells with DSS treatment showed a significant increase in the number of colonies compared to that in CRC cells treated with DSS or *F. nucleatum* alone (Figure 1D,E). These results suggested that the presence of *F. nucleatum* in CRC cells affected cellular growth by promoting the proliferative activity of cells rather than having a cytotoxic effect.

### 2.2. F. nucleatum Increases the Motility of CRC Cells Under DSS Stimulation by Promoting Epithelial-to-Mesenchymal Transition (EMT)

**Figure 2:**
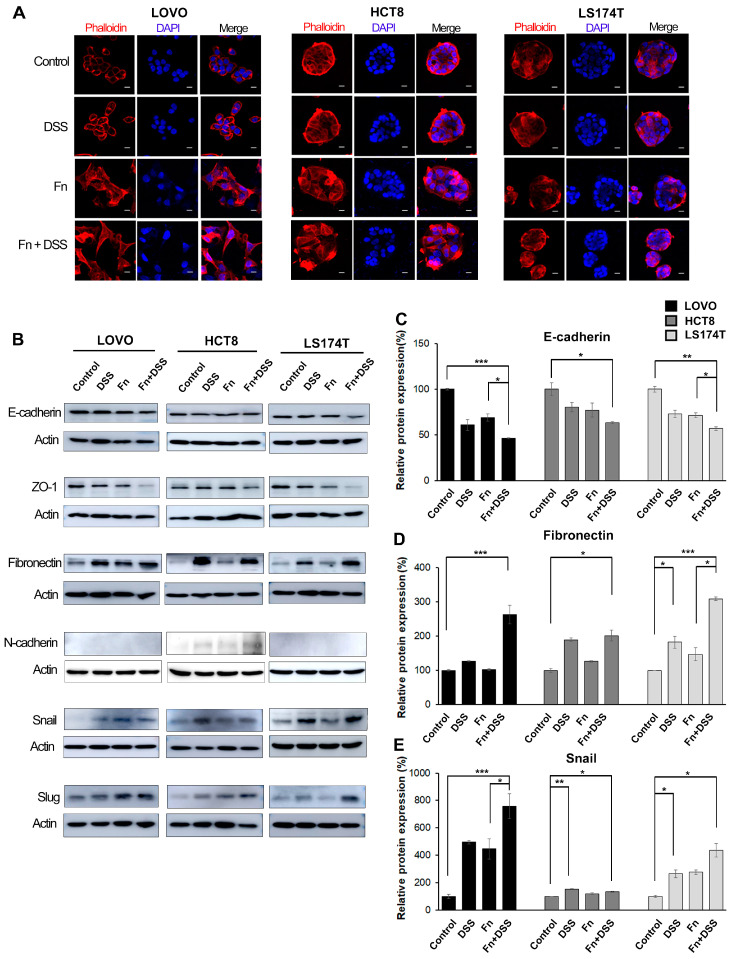
*F. nucleatum* induces morphological changes in CRC cells by promoting epithelial–mesenchymal transition (EMT). (**A**) Immunofluorescence analysis showing the morphology of CRC cells treated with vehicle, DSS, *F. nucleatum*, or DSS + *F. nucleatum*. Cells were stained with rhodamine-phalloidin (red) to label filamentous actin and with DAPI (blue) to label the nucleus; 200× magnification; scale bar: 10 μm. (**B**) Western blot analysis of EMT-associated factors in CRC cells. Uncropped Western Blots can be observed in Figure S6. (**C**–**E**) The graph represents relative protein levels obtained from three independent experiments. Densitometry of blots was followed by the calculation of relative protein levels compared with the control after normalization to beta-actin levels. The data are presented as the mean ± SEM (* *p* < 0.05, ** *p* < 0.01, and *** *p* < 0.001; unpaired Student’s *t*-test). Abbreviations: DSS, dextran sodium sulfate; Fn, *F. nucleatum*.

To determine the morphologic changes in CRC cells induced by *F. nucleatum*, cells were infected with *F. nucleatum* for 24 h and then fixed, stained with fluorescent TRITC-conjugated phalloidin, and imaged using a confocal microscope. After infection with *F. nucleatum*, LOVO and HCT8 cells lost their epithelial cell morphology and displayed an elongated shape, which is a typical mesenchymal morphology (Figure 2A). When LOVO and HCT8 cells were treated with both *F. nucleatum* and DSS, the cell-cell junctions completely disintegrated, thereby enhancing their cell migratory ability compared to cells treated with *F. nucleatum* or DSS alone. In contrast to the two other cell lines, LS174T cells exhibited no prominent morphological changes following infection with *F. nucleatum*, but they produced many small cell nests (Figure 2A). These results indicated that *F. nucleatum* caused LS174T cells to scatter and then grow by forming many small nests. As observed in Figure 2A, the change in CRC cell morphology to a slender shape is one of the basic features of epithelial–mesenchymal transition (EMT). The alteration of EMT in CRC cells induced by *F. nucleatum* was further verified by assessing major EMT-associated markers and transcription factors using Western blot analysis (Figure 2B). *F. nucleatum* significantly decreased the expression of epithelial markers, such as E-cadherin and ZO-1 (Figure 2B,C), and it increased the expression of mesenchymal phenotype-associated molecules, such as fibronectin and N-cadherin (Figure 2B,D). *F. nucleatum* infection did not affect vimentin and alpha-sma expression. In addition, we found that *F. nucleatum* increased EMT-associated transcription factors such as Snail and Slug (Figure 2B,E). The other transcriptional factors that were assessed, Zeb1 and Twist, were not induced by *F. nucleatum*. The treatment of CRC cells with both *F. nucleatum* and DSS synergistically changed the expression of EMT molecules compared to that in CRC cells treated with DSS or *F. nucleatum* alone (Figure 2B). In a preliminary study, *F. nucleatum* infection decreased E-cadherin and increased Snail and Slug in LOVO cells in a dose-dependent manner, whereas *P. gingivalis* infection had no effect on EMT-associated molecules (Figures S2 and S10).

**Figure 3:**
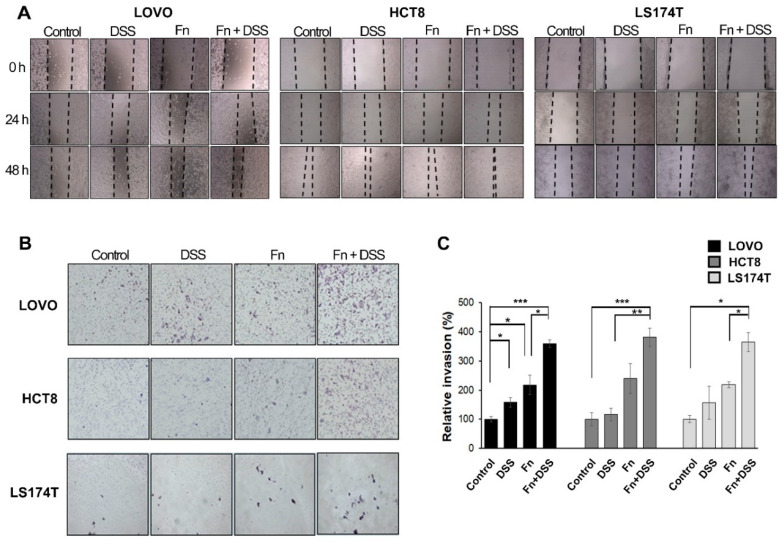
*F. nucleatum* increases the motility of CRC cells under DSS stimulation. (**A**) Wound-healing assay of CRC cells co-cultured with vehicle, DSS, *F. nucleatum*, or *F. nucleatum* + DSS and monitored for 48 h with 24 h intervals. 40 times magnification. (**B**) Transwell-invasion assays were conducted with CRC cells co-cultured with vehicle, DSS, *F. nucleatum*, or *F. nucleatum* + DSS. 100 times magnification. (**C**) Graph showing the percentage of invaded CRC cells. The indicated invaded cells were quantified in five randomly selected fields. The graph represents relative levels from three independent experiments. The data are presented as the mean ± SEM (* *p* < 0.05, ** *p* < 0.01, and *** *p* < 0.001; unpaired Student’s *t*-test). Abbreviations: DSS, dextran sodium sulfate; Fn, *F. nucleatum*.

EMT modulates the biological behaviors of cancer cells, such as their invasive and high motility properties [16,17,18]. Increases in the migratory and invasive abilities of CRC cells following *F. nucleatum* infection were observed in wound-healing and transwell-invasion assays (Figure 3). A wound-healing assay was performed to characterize the migration of *F. nucleatum*-infected CRC cells for 24 and 48 h (Figure 3A). Closure of the wound area was faster for *F. nucleatum*-infected CRC cells under DSS treatment than for CRC cells treated with DSS or *F. nucleatum* alone. We investigated the effect of *F. nucleatum* on the invasive ability of CRC cells using a transwell invasion assay (Figure 3B). CRC cells infected with *F. nucleatum* showed a two-fold increase in invasion, and the combination of *F. nucleatum* infection and DSS further enhanced invasion ability (Figure 3C). There was no significant change in the invasive ability of CRC cells following treatment with DSS alone (Figure 3C). These results indicated that *F. nucleatum* infection increased the motility of CRC cells compared to that of the non-infected cells.

**Figure 4:**
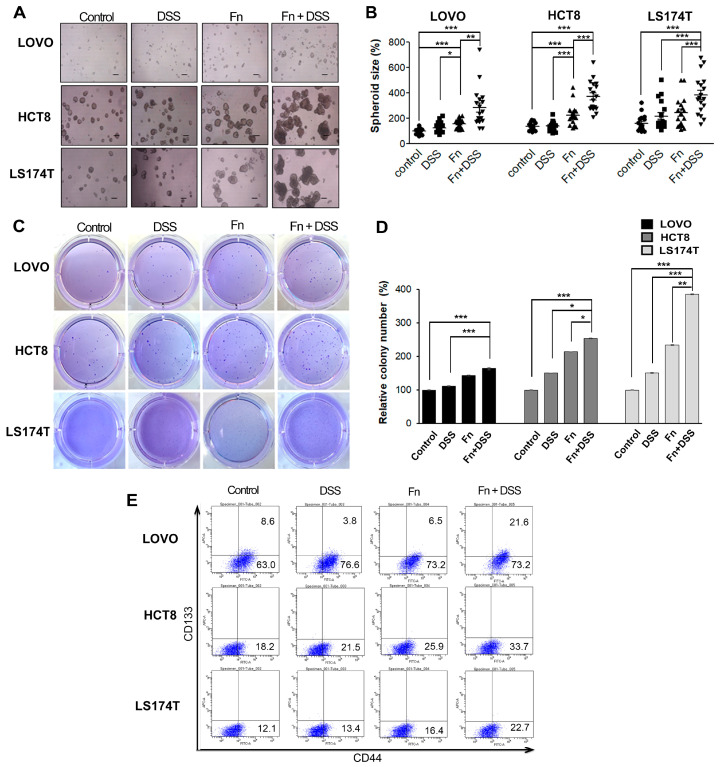
*F. nucleatum* enhances the stemness of colorectal cancer cells. (**A**) Effects of *F. nucleatum* on the sphere formation ability of CRC cells. CRC cells (LOVO, HCT8, and LS174T) were subjected to a sphere formation assay for 3 d after treatment with vehicle, DSS, *F. nucleatum*, or *F. nucleatum* + DSS. Representative images of spheres of CRC cells are shown. Scale bar, 200 μm. (**B**) Graphs presenting the mean diameters of spheres. (**C**) Colony-formation ability of CRC cells in a soft agar assay. (**D**) Graph presenting the mean diameters of spheres. (**E**) Expression profiles of CD133 and CD44 in CRC cell lines characterized using flow cytometry. Cell suspensions were labeled with PE-conjugated anti-CD133 and FITC-conjugated anti-CD44 antibodies and analyzed using a flow cytometer. Isotypic controls were used to establish the right gating. The number of double-positive cells in the right top quarter is expressed as a percentage of total cells. The graph represents relative levels from three independent experiments. The data are presented as the mean ± SEM (* *p* < 0.05, ** *p* < 0.01, and *** *p* < 0.001; unpaired Student’s *t*-test). Abbreviations: DSS, dextran sodium sulfate; Fn, *F. nucleatum*.

Cancer stemness (CS) along with EMT is known to be central to inducing metastasis in various cancers [19,20,21]. We hypothesized that *F. nucleatum* would enhance the stemness of CRC cells. To test this hypothesis, we used both sphere-formation assays and an anchorage-independent growth assay in soft agar. *F. nucleatum* increased the size of spheroids in LOVO and HCT8 cells compared to that in the control, and the combination of *F. nucleatum* infection and DSS further promoted the sphere-formation ability (Figure 4A,B). In addition, the colony formation ability of CRC cells was also assessed using a soft agar assay. As shown in Figure 4C,D, *F. nucleatum*-infected CRC cells formed a significantly higher number of colonies than uninfected cells, and the combination of *F. nucleatum* infection and DSS further promoted the colony-formation ability. Then, we determined whether *F. nucleatum* infection increased the expression of stem cell-related markers, such as CD44 and CD133, in CRC cells using flow cytometry. *F. nucleatum*-infected CRC cells exhibited a high expression of CD44, but there was no change in the expression of CD133 (Figure 4E, Table 1). The combination of DSS and *F. nucleatum* infection further promoted the expression of CD44 in CRC cells compared to that in CRC cells treated with DSS or *F. nucleatum* alone (Figure 4E, Table 1). These results indicated that *F. nucleatum* infection increased the motility and stemness of CRC cells by promoting EMT.

### 2.3. F. nucleatum Accelerates the Progression of CRC by Promoting EMT in an AOM/DSS Mouse Model

**Figure 5:**
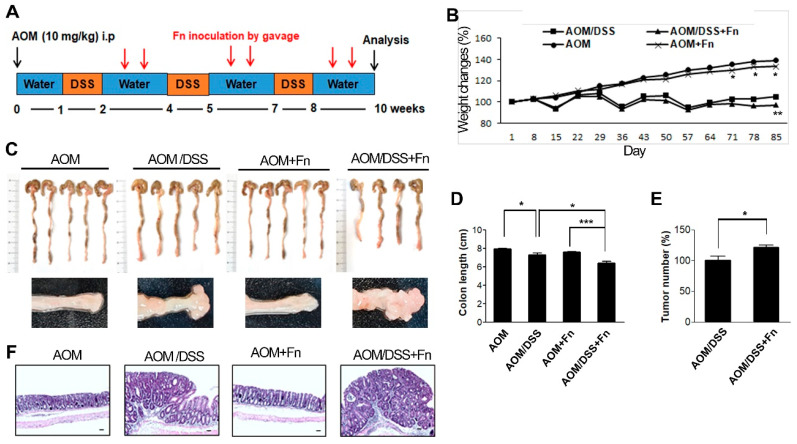
*F. nucleatum* accelerates the progression of CRC in AOM/DSS model mice. Tumors were induced in C57BL/6 mice with AOM/DSS. (**A**) Experimental procedure for the establishment of the ulcerative colitis-related colorectal cancer model and treatment. Abbreviations: i.p. injection, intraperitoneal injection. (**B**) Body weight changes in mice were monitored at the indicated times after AOM injection. The data are presented as the mean ± SEM (* *p* < 0.05, and ** *p* < 0.01; unpaired Student’s *t*-test). (**C**) Representative morphological features of the whole colons from mice with or without *F. nucleatum* infection in the AOM/DSS model (upper panel). Lower panels are enlarged images. (**D**) Colon length was measured on day 85. The data are presented as the mean ± SEM (* *p* < 0.05 and *** *p* < 0.001; unpaired Student’s *t*-test). (**E**) The number of tumor nodules in colon tissue was counted on week 12. Data are presented as the average tumor number ± SEM (*n* = 6). * *p* < 0.05 compared with the AOM/DSS group. (**F**) Representative hematoxylin and eosin-stained sections of the distal colon showing the morphology of AOM, AOM/DSS, AOM + *F. nucleatum*, or AOM/DSS + *F. nucleatum*-treated mice (*n* = 5 per group). 100 times magnification; scale bar: 100 μm. Abbreviations: AOM, azoxymethane; DSS, dextran sodium sulfate; Fn, *F. nucleatum*.

**Figure 6:**
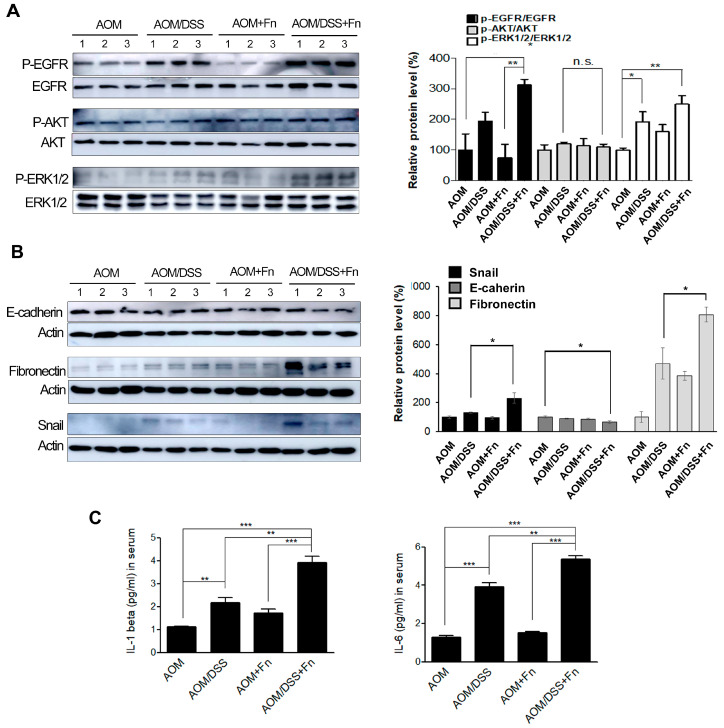
*F. nucleatum* triggers EMT of CRC cells in an AOM/DSS mouse model. (**A**) Phosphorylation levels of epidermal growth factor receptor (EGFR), protein kinase B (AKT), and extracellular signal-regulated kinase 1/2 (ERK1/2) in colonic tissues from each group were determined using Western blot analysis. Abbreviations: n.s, non significant. The Uncropped Western Blots are available in Figure S7. (**B**) The expression levels of Snail, E-cadherin, and fibronectin in colonic tissues from each group were determined using Western blot analysis. Graphs of the results of the analyses of the indicated proteins. * *p* < 0.05 compared to the AOM/DSS group. The graph represents relative protein levels obtained from three independent experiments. Densitometry of blots was followed by the calculation of relative protein levels compared with the control after normalization to beta-actin levels. (**C**) Effect of *F. nucleatum* on the levels of pro-inflammatory cytokines. The levels of interleukin IL-1 beta and IL-6 in serum from each group. The data are presented as the means ± SEM (** *p* < 0.01 and *** *p* < 0.005; unpaired Student’s *t*-test). The number of each group is 5. Abbreviations: AOM, azoxymethane; DSS, dextran sodium sulfate; Fn, *F. nucleatum*.

To investigate the effects of *F. nucleatum* on the tumorigenesis of CRC, we established an AOM/DSS model in C57BL/6 mice (Figure 5A). After inducing mutagenesis with AOM, DSS was administered to the mice in three repeated rounds, and the mice were inoculated with *F. nucleatum* twice after each of the three rounds of DSS administration for a total of six inoculations (Figure 5A). The conditions and body weights of the mice were monitored until the end of the experiment. The AOM/DSS mice exhibited fluctuations in body weight compared to the AOM mice (Figure 5B). *F. nucleatum* infection exacerbated the body weight loss at 78 and 85 d in the AOM/DSS mice but did not affect weight loss in the AOM mice (Figure 5B). As shown in Figure 5C,D, the colon length was shorter in the AOM/DSS + *F. nucleatum*-treated mice than in the mice in the other groups. The combination of AOM/DSS and *F. nucleatum* increased the incidence of colonic neoplasm compared to that in the AOM/DSS mice (Figure 5C,E). No colon tumors were observed in the AOM and AOM + *F. nucleatum* groups (Figure 5C,E). The AOM/DSS mice exhibited bloody diarrhea and spleen hypertrophy owing to severe inflammation, and *F. nucleatum* further exacerbated these symptoms (Figures S3 and S4). Histological examination of the colonic sections was performed to assess the extent of histopathological changes and severity of dysplasia. Dysplasia was generally worse in the *F. nucleatum*-treated AOM/DSS mice than in the untreated AOM/DSS mice (Figure 5F). An inflammatory response occurs when tissues are damaged by bacteria, toxins, or various external stimuli. To determine whether inflammatory factors were also induced by *F. nucleatum* infection, we measured the serum levels of pro-inflammatory cytokines. Interestingly, we found that several inflammatory factors, including IL-1β and IL-6, were significantly upregulated in the *F. nucleatum*-treated AOM/DSS group (Figure 6C).

To determine whether EMT contributes to the pathogenesis of colitis-associated colorectal cancer, the expression of EMT-related genes, such as Snail, E-cadherin, and fibronectin, in colon lysates was analyzed. The AOM/DSS/Fn group enhanced the phosphorylation of epidermal growth factor receptor (EGFR) and its downstream target proteins, protein kinase B (AKT) and extracellular signal-regulated kinase (ERK), compared to the AOM/DSS group (Figure 6A). EMT induction by AOM/DSS was significantly accelerated by *F. nucleatum* treatment (Figure 6A,B). The expression of Snail and E-cadherin in the colon tumor mass also was assessed using immunohistochemistry, and the results showed a trend similar to those seen in the Western blot analysis (Figure S4). The expression of Snail, E-cadherin, and fibronectin was significantly correlated with *F. nucleatum* infection, suggesting the potential involvement of *F. nucleatum* in CRC progression.

### 2.4. F. nucleatum Stimulates EMT through Epidermal Growth Factor Receptor (EGFR) Activation

**Figure 7:**
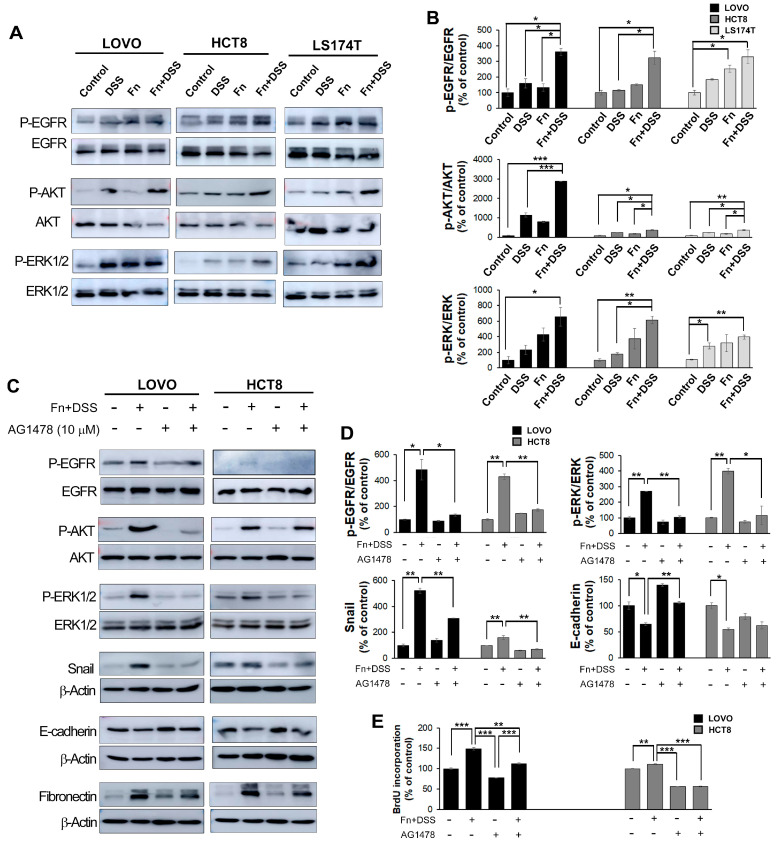
*F. nucleatum* promotes EMT through the EGFR signaling pathway. (**A**) The expression of EGFR pathway-related markers in CRC cells co-cultured with vehicle, DSS, *F. nucleatum*, or *F. nucleatum* + DSS was analyzed using Western blotting. Uncropped Western Blot Figures are available in Figure S8. (**B**) Quantitation of (**A**). Band intensities of each target protein were measured using an image analyzer and expressed as relative ratios. Data are the mean ± SEM. * *p* < 0.05 and *** *p* < 0.005 vs. corresponding controls. (**C**) The expression of EGFR pathway-related markers and EMT-related markers in CRC cells with or without AG1478 was analyzed using Western blotting. Uncropped Western Blot Figures are available in Figure S9. (**D**) Quantitation of (**C**). The band intensities of each target protein were measured using Image J software (ImageJ bundled with 64-bit Java 1.8.0_172, web site: https://imagej.nih.gov/ij/index.html) and expressed as relative ratios. (**E**) The graph represents relative protein levels obtained from three independent experiments. Densitometry of blots was followed by the calculation of relative protein levels compared with the control after normalization to beta-actin levels. Data are the mean ± SEM. * *p* < 0.05, ** *p* < 0.01 and *** *p* < 0.005 vs. corresponding controls. Abbreviations: AOM, azoxymethane; DSS, dextran sodium sulfate; Fn, *F. nucleatum*.

EMT is regulated by various signaling mechanisms, including cell surface receptor tyrosine kinases, and the associated interactions are important for its induction [22,23]. Epidermal growth factor receptor (EGFR) is one of the tyrosine kinases related to EMT [24]. EGFR is known to induce the activation of downstream effector kinases, including protein kinase B (AKT) and extracellular signal-regulated kinase (ERK) [25]. Thus, to determine whether the co-treatment of CRC cells with *F. nucleatum* and DSS affects EGFR, AKT, and ERK, the phosphorylated levels of EGFR, AKT, and ERK were assayed using Western blotting. As expected, the combined treatment with *F. nucleatum* and DSS enhanced the phosphorylation of these proteins compared to the levels in the other groups; however, it had no effect on the levels of total EGFR, and it decreased the total levels of AKT and ERK (Figure 7A,B).

Next, CRC cells were treated with an EGFR inhibitor (AG1478, Calbiochem, La Jolla, CA, USA) to determine whether EGFR was required for the induction of EMT by the *F. nucleatum* and DSS co-treatment. We found that AG1478 markedly reduced the EGFR phosphorylation induced by *F. nucleatum* and DSS co-treatment compared to that in the non-treated group, suggesting that AG1478 eliminated EGFR activation. The expression of EGFR downstream genes containing AKT and ERK directly was downregulated in the presence of the EGFR inhibitor. Moreover, EGFR inhibition significantly reduced the EMT transcription factor Snail and induced the expression of the epithelial marker E-cadherin in *F. nucleatum*- and DSS-treated CRC cells (Figure 7C,D).

Collectively, our results suggested that the induction of EMT by *F. nucleatum* could increase the motility and the stemness of CRC cells and consequently promote colon tumorigenesis.

## 3. Discussion

*F. nucleatum*, a Gram-negative anaerobic bacterium, is one of the pathogens that cause periodontitis; it has been shown to be an important driver in both UC and CRC [26,27]. Previous studies have reported that *F. nucleatum* contributes to the progression of colitis by damaging the integrity of colon epithelium and downregulating intercellular adhesion molecules, and that it also induces characteristic changes in macrophages in the colorectal tumor microenvironment [28]. Studies on the development and progression of CRC have shown that *F. nucleatum* promotes tumor formation as well as chemoresistance [29]. Although numerous works have reported the importance of *F. nucleatum* in the pathogenesis of UC and CRC, the role of *F. nucleatum* in the pathogenesis of CAC [30], which is the product of the close relationship between UC and CRC, remains unclear.

In the present study, we determined whether *F. nucleatum* could modify the EMT and development of CAC. We found that *F. nucleatum* accelerated the growth and tumorigenic potential of colitis-associated CRC cells, as evidenced by a higher proliferation rate and colony-forming ability in *F. nucleatum*-infected CRC cells with DSS treatment compared to those in cells treated with DSS or *F. nucleatum* infection alone. In addition, FN-infected/DSS-treated CRC cells formed more and larger tumor spheroids than cells treated with FN infection or DSS alone. These findings suggest the possibility that *F. nucleatum* infection plays an important role in the progression of CAC. The enhanced oncogenic potential of CAC following *F. nucleatum* infection was further supported by an in vivo experiment in an AOM/DSS-induced CAC mouse model, in which there was a larger number of tumor nodules in the group treated with AOM/DSS and *F. nucleatum* inoculation than in the group treated with AOM/DSS alone.

Among the numerous ways in which cancer cells display biological behaviors, including invasion and metastasis, EMT, a process involving the transition from an epithelial to a mesenchymal phenotype, is the most well-known [31]. The prognostic importance of EMT markers was analyzed using Kaplan–Meier survival curves and a human protein atlas program. Based on the Kaplan–Meier survival curves, patients with high Snail expression had a significantly lower survival probability than those with low Snail expression, while the opposite was true for E-cadherin (Figure S5). These results indicated that CRC was positively associated with Snail expression but negatively associated with E-cadherin expression. Previous studies on the role of *F. nucleatum* in the progression of CRC reported that *F. nucleatum* accelerates the metastatic potential of CRC and that the presence of *F. nucleatum* is related to the advanced stages of CRC, suggesting that the increased aggressiveness of CRC induced by *F. nucleatum* possibly depends on EMT. However, there have been few studies on EMT induction by *F. nucleatum*, and only a very recent study showed a correlation between *F. nucleatum* and metastasis [32].

Although the effect of DSS on EMT has not been extensively studied, a study that investigated DSS-induced intestinal fibrosis found that DSS induces the expression of EMT-like features in mice, including reducing E-cadherin and significantly inducing Snail [33]. Here, we found that *F. nucleatum* promoted the aggressiveness of CRC and CAC cells based on the increased migratory and invasive capabilities in FN-infected/DSS-treated CRC cells and FN-infected cells. Furthermore, DSS and *F. nucleatum* synergistically modulated the expression of EMT-related transcription factors and EMT markers in both in vitro and in vivo experiments, indicating that the increased aggressiveness of CAC induced by *F. nucleatum* is acquired through promoting EMT. Notably, *F. nucleatum* has been suggested to be a risk factor mainly in CRC, while *Porphyromonas gingivalis*, another major periodontopathic bacterium, has been extensively studied as an important pathogen in the pathogenesis of other types of cancers such as oral, esophageal, and pancreatic carcinomas. *P. gingivalis* has been reported to play a role in EMT in a few studies on oral cancer [34]. In Figure 7A, protein expression of AKT and ERK tended to decrease in the DSS + *F. nucleatum* group. The degradation of AKT and ERK can be due to ubiquitination, caspase cleavage, and other factors. The decrease in protein expression of AKT and ERK is thought to be degradation due to toxicity to DSS and *F. nucleatum* depending on the cell condition. It is assumed that the activation of EGFR signals by *F. nucleatum* is possible both in the cell membrane and inside the cytoplasm. In general, it is expected that oncogenic signals are activated by LPS secreted by *F. nucleatum*. However, it remains uncertain how directly virulence is linked to host cell invasion. New studies have shown that *F. nucleatum* can bind to host colon cells and stimulate directly oncogenic signaling by interactions between fusobacterial FadA and host E-cadherin [35]. It is presumed that *F. nucleatum* within the cytoplasm directly and indirectly (*F. nucleatum*-secreting LPS) activates carcinogenic signals. We believe that EMT promotion through EGFR activation by *F. nucleatum* may have a mechanism similar to other carcinogenic signals. In this study, we found no changes in the levels of EMT-related factors in *P. gingivalis*-infected CRC cells (data not shown), while *F. nucleatum* dramatically affected EMT-related molecules. In addition, EMT is known to be closely associated with stemness properties, and these two phenomena are intertwined [36]. FN-infected/DSS-treated CRC cells were found to have the strongest stemness characteristics, indicating that *F. nucleatum* promotes the aggressiveness of CAC via increasing stemness as well as EMT. Since EMT is possibly a major phenomenon that leads to a poor prognosis for patients with cancer, we investigated ways to reverse the EMT that is induced by *F. nucleatum* infection and DSS treatment. The EGFR signaling pathway has been reported to be one of the mechanisms by which EMT-like features are induced in various cancers. A recent study on salivary gland cancer revealed that EGFR activation induces EMT in a Snail-dependent manner, and a study using CRC cells found a high expression of EGFR and the occurrence of EMT following EGFR activation through EGF binding to EGFR [37]. In this study, we observed the activation of EGFR, Akt, and Erk, an increase in EMT-related transcription factors and markers in DSS-treated cells also infected with *F. nucleatum*, and an EGFR inhibitor that modulated the EMT program by inactivating these kinases. Taken together, these findings suggested that *F. nucleatum* is a novel predictive biomarker for EMT, and targeting *F. nucleatum* may be an effective approach to prevent the development of CRC.

## 4. Materials and Methods

### 4.1. Cell Culture

Three colon cancer cell lines (LOVO, HCT8, LS174T) were used in this study. LOVO cells were grown in Harm’s F12K medium (Welegene, Daegu, Korea), LS174T cells (ATCC, Manassas, VA, USA) were grown in MEM (Welegene, Daegu, Korea), and HCT8 cells (ATCC, Manassas, VA, USA) were grown in RPMI 1640 medium (Welegene, Daegu, Korea); all media contained 10% fetal bovine serum (FBS; Hyclone, South Logan, UT, USA), and all cells were grown at 37 °C in a humidified environment with 5% CO_2_ and 95% O_2_. For this study, the CRC cells were infected with live *F. nucleatum* at a MOI (multiplicity of infection) of 1:500 for 4 h at 37 °C in 5% CO_2_. Then, the cells were washed with phosphate-buffered saline (PBS, Welegene, Daegu, Korea) and the media was replaced with fresh media containing 2.5% 50,000 Da DSS (MP bio, Santa Ana, CA, USA) until the cells were harvested. Controls were subjected to similar media changes and wash conditions but without bacterial inoculation.

### 4.2. Bacterial Culture and Bacterial Internalization into CRC Cells

*F. nucleatum* was cultured anaerobically and grown in a GAM broth (Nissui Pharmaceutical, Tokyo, Japan) at 37 °C overnight. Colon cancer cells were infected with the CFSE-labeled bacteria at an MOI of 500 for 3 h. For confocal microscopy, the infected cells were fixed, permeabilized, and then stained with rhodamine–phalloidin (Invitrogen, Taastrup, Denmark) and DAPI (Invitrogen). Mounted slides were imaged using a confocal microscope (Carl Zeiss, Oberkochen, Germany).

### 4.3. Cell Proliferation Assay

Cell proliferation was performed using MTT assay by an EZ-Cytox cell viability assay kit (Daeillab service, Seoul, Korea) and a BrdU proliferation assay kit (Calbiochem, San Diego, CA, USA) according to the manufacturer’s protocols. The results are expressed as percentages after comparing the absorbance at 450 nm with that in the uninfected control cells.

### 4.4. Western Blot Analysis

To analyze protein expression, Western blotting was performed using specific antibodies. Briefly, the cells were washed once with cold PBS and lysed with 1X RIPA buffer (Cell signaling Technology, Danvers, MA, USA) containing a protease inhibitor cocktail and phosphatase inhibitors. The lysates were centrifuged at 13,000× *g* for 10 min. Subsequently, the supernatants were collected. Using the Bradford method, the protein concentration was determined to be 20 µg. Samples were separated on 8–12% SDS-polyacrylamide gels (Bio-Rad, Hercules, CA, USA). Then, the SDS-gels were transferred electrophoretically onto polyvinylidene fluoride membranes (Bio-Rad, Hercules, CA, USA) using a wet transfer kit. The transferred membranes were blocked with 5% skim milk in TBST (BD Biosciences, San Jose, CA, USA) containing 0.1% Tween-20 (Bio-Rad, Richmond, CA, USA) for 1 h at room temperature. The membranes were incubated overnight at 4 °C with the primary antibodies against E-cadherin (Cell Signaling, #3195, Danvers, MA, USA), fibronectin (BD Bioscience, #610077 San Jose, CA, USA and Santa Cruz, #SC-9068, CA, USA), Snail (Cell Signaling, #3879, Danvers, MA, USA), p-EGFR (Invitrogen, #18-2463, Carlsbad, CA, USA), EGFR (BD Bioscience, Santa Cruz, #SC-03, CA, USA), p-AKT (Cell Signaling, #9271, Danvers, MA, USA), AKT (Cell Signaling, #9272, Danvers, MA, USA), p-ERK1/2 (BD Bioscience, Santa Cruz, #SC-7976, CA, USA), ERK1/2 (BD Biosciences, Santa Cruz, #SC-94, CA, USA), and β-actin (BD Bioscience, Santa Cruz, #SC-47778, CA, USA). The membranes were washed three times with 1x TBST buffer, and HRP-conjugated secondary antibodies (ENZO, #ADI-SAB-300-J, and #ADI-SAB-100-J, 1:8000, NY, USA) were applied for 2 h at room temperature. The antigen-antibody complexes were detected using a SuperSignal West–Femto reagent (Thermo Fisher, Waltham, MA, USA).

### 4.5. Colony Formation Assay

For the colony formation assay, single cells (1000 cells/well) were plated in 6-well plates. The cells were allowed to grow for 7–11 d, and the media was changed every 2 d. The colonies were fixed with 4% paraformaldehyde for 30 min. After washing three times with PBS, colonies were stained with 0.1% crystal violet (Sigma-Aldrich, St. Louis, MO, USA) for 15 min. After they were washed with distilled water, the cells were photographed and analyzed to assess their proliferation and colony-forming efficiency. All experiments were performed at least two times.

### 4.6. Colon Spheroid Formation

Colon cancer cells were grown as spheroids. Briefly, single-cell suspensions were seeded in 24-well culture plates at a density of 5 × 10^3^ cells/well in a tumor sphere medium (medium supplemented with B27 (Invitrogen, Karlsruhe, Germany) containing 10% FBS), pre-coated with a poly-2-hydroxyethyl methacrylate (polyhema) solution (Sigma-Aldrich, St. Louis, MO, USA). The spheroids were incubated for 3–6 days; then, the spheres in each well were counted. The total number of tumor spheroids formed in each well was plotted, and representative images were taken.

### 4.7. Soft Agar Assay

Treated cells were harvested and pipetted to form a single-cell suspension. The cells were seeded at a density of 1.5 × 10^3^–3 × 10^3^ cells/well in growth medium containing 0.25% soft agar (top layer) on top of 0.5% soft agar (base layer) in 6-well plates and cultured for an additional 2–3 weeks. The colonies that appeared were observed using microscopy, and the colony number was determined after staining with 0.05% (*w*/*v*) crystal violet (in 5% methanol).

### 4.8. Wound-Healing Assay

Wound-healing assays were performed to evaluate cell-migration ability. Briefly, colon cancer cells were seeded at a density of 1 × 10^6^ cells/mL in 6-well plates and incubated in FBS-containing medium. When they reached 80% confluence, the cells were infected with *F. nucleatum* with the addition of 1 mM thymidine (Sigma-Aldrich, St. Louis, MO, USA) for the indicated times. After 4 h, a wound was made in the middle of the well using a sterile 200 µL pipette tip. The wells were washed three times with PBS; then, the colon-cancer cells were incubated with media containing 1% FBS and 2.5% DSS for 24 h. Images were captured using a microscope (Nikon Eclipse TS100, Tokyo, Japan). The rates of the healing of the wound area were calculated as a percentage of the remaining wound area compared to the initial wound area.

### 4.9. Transwell Invasion Assay

Cell invasion capacity was assessed using 8 µm pore size 24-insert transwell chambers (Corning, NY, USA). For the invasion assay, the upper chambers were pre-coated with Matrigel (BD Biosciences, San Jose, CA, USA). Medium containing 10% fetal bovine serum (700 µL) was added to the lower chamber. After 48 h, cells on the bottom of the inserts were fixed in 4% paraformaldehyde and stained with hematoxylin (YD CORP., Gyeonggi-do, Korea) and eosin (Sigma-Aldrich, St Louis, MO, USA). Then, cells that invaded the lower surface were counted in at least three fields using microscopy (Nikon Eclipse TS100, Tokyo, Japan). Each experiment was performed at least two times.

### 4.10. Flow Cytometry

The expression of the CSC markers CD133 and CD44 was detected using flow cytometry. Briefly, colon cancer cells were harvested with 0.05% trypsin and washed with phosphate-buffered saline (PBS). Cells were incubated with FITC mouse anti-human CD44 (BD Biosciences, Franklin Lakes, NJ, USA) and CD133/2-APC human antibodies (Miltenyi Biotec, Bergisch Gladbach, Germany) in the dark at 4 °C. After 30 min, the cells were washed and analyzed using an FACS system (BD, San Jose, CA, USA).

### 4.11. Animals and Establishment of an AOM/DSS Mouse Model

Female C57BL/6 mice (6 weeks old) were purchased from Orient Bio Inc (Seungnam, Korea). The care of animals and experimental procedures were approved by the Institutional Animal Care and Use Committee of Pusan National University (Protocol No. PNU-2019-2207, June 2019). All animals were housed under controlled SPF conditions (temperature 22 ± 1 °C, 12 h dark/light cycle) with free access to a standard diet and water. The procedure used for the establishment of the UC-CRC model using AOM and DSS is shown in Figure 5A. The mice were randomly divided into four groups: the AOM group (*n* = 5), AOM/DSS group (*n* = 5), AOM + Fn group (*n* = 5), and AOM/DSS + Fn group (*n* = 5). To develop the UC-CRC model, 7-week-old female C57BL/6 mice were given a single intraperitoneal injection of azoxymethane (Sigma-Aldrich, St.Louis, MO, USA) at a dose of 10 mg/kg. One week later, the animals were given 2.5% DSS in their drinking water for 7 d followed by 14 d of normal drinking water for recovery, and this cycle was repeated three times. *F. nucleatum* (1 × 10^9^/CFU) or the vehicle was ad, ministrated by gavage six times until the end of the 12-week study period. During the experimental period, body weights were measured every week. At the end of the experiment, blood was collected for ELISA, the mice were sacrificed, and colon tissues were removed. After the weights and lengths of the tissues were measured, the colons were slit open longitudinally along the main axis and washed with PBS (pH 7.4). The number of tumors in the colons was recorded. Subsequently, some colon tissues were fixed in 4% paraformaldehyde buffer for further histopathological examination and immunohistochemical analysis, while others were flash-frozen in liquid nitrogen and kept at −80 °C for Western blot analysis.

### 4.12. Hematoxylin and Eosin (H & E) Staining

Large intestine tissue samples were fixed in 4% paraformaldehyde solution and embedded in paraffin. For histopathology analysis, paraformaldehyde-fixed colonic tissues were dehydrated in a gradient alcohol series, embedded in paraffin, and cut into 5 μm-thick serial sections. Then, tissue sections were stained with H & E to examine tissue morphology and observed using optical microscopy.

### 4.13. Immunohistochemistry

Tissue samples were dehydrated, embedded in paraffin, and cut into 5 μm-thick sections for immunohistochemistry (IHC). Prior to being subjected to immunostaining, 4% paraformaldehyde-fixed paraffin-embedded tumors were excised from mouse flanks (5 μm), deparaffinized, and rehydrated. Tissue slides were heated in 10 mM sodium acetate (pH 6.0, Duksan Chemical Co., Seoul, Korea) for 10 min at 121 °C for antigen retrieval and then bathed in a 0.3% H_2_O_2_-PBS solution (Welegene, Deagu, Korea) for 15 min at room temperature in the dark to quench endogenous peroxidase. After samples were washed with 0.5% Tris-HCl-Tween (Welegene, Deagu, Korea), tissue sections (5 μm) were blocked with 5% FBS (Hyclone, South Logan, UT, USA) in PBS (Welegene, Deagu, Korea) with 0.1% Tween 20 (PBST), incubated in a 1:250 dilution of the primary antibodies overnight at 4°C, and repeatedly washed with PBST. Samples were incubated with horseradish peroxidase-conjugated secondary antibody (ENZO, #ADI-SAB-300-J, 1:250, New York, NY, USA) for 2 h at room temperature, after which they were subjected to repeated washing with PBST. Then, the bound antibodies were determined using freshly prepared substrate buffer (0.05% diaminobenzidine (DAB; Sigma-Aldrich), 0.015% H_2_O_2_ in PBS) for 2 min. After a final wash in PBS and distilled water, the slides were counterstained with Mayer’s hematoxylin (MUTO, Tokyo, Japan) for 4 min and then dehydrated in a graded alcohol series (50%, 70%, 80%, 90%, 95%, 100%, and 100%, Samjun, Seoul, Korea). Sections were subsequently examined at various magnifications using fluorescence microscopy (OLYMPUS, Tokyo, Japan).

### 4.14. Fusobacterium Nucleatum-Specific Detection Using RNA Extraction and Real-Time PCR

Total RNA was extracted from cells using an RNeasy Mini kit (Qiagen, Hilden, Germany) according to the manufacturer’s instructions. The isolated DNA was used as a template and detected using *F. nucleatum* 16s rRNA-specific primers. *F. nucleatum* 16s rRNA was detected using two-step quantitative real-time PCR with a QuantiTect reverse transcription kit (Qiagen, Hilden, Germany) and TOPreal SYBR Green PCR Kit (Enzynomics, Seoul, Korea) in an ABI 7500 real-time PCR detection system (Applied Biosystems, Foster City, CA, USA). Reactions were carried out for 45 cycles. Reactions were duplicated, and the average mRNA level of each gene was determined using the 2-ΔΔCt method. The following primers were used for real-time PCR: 5′-GGG-CTC-AAC-TCT-GTA-TTG-CG-3′ and 5′-CTG-TTT-GCT-ACC-CAC-GCT-TT-3′.

### 4.15. Cytokine Analysis

Blood was retro-orbitally collected to measure cytokines. Serum was separated using centrifugation and stored at − 80 °C until measured. Samples were diluted 1:1, and serum IL-1β and IL-6 pre-inflammatory cytokine levels were measured using Affymetrix mouse ELISA kits (Invitrogen, San Diego, CA, USA) according to the manufacturers’ instructions.

### 4.16. Statistical Analyses

Statistical analyses were performed using GraphPad Prism version 5.01 (GraphPad Software, Inc., San Diego, CA, USA, website: https://www.graphpad.com/scientific-software/prism/). For the comparative analysis of two groups of data, Student’s *t*-test was performed. *p*-values < 0.05 were considered statistically significant. All data are shown as the mean and standard error of the mean (SEM).

## 5. Conclusions

This study examined the effects of *F. nucleatum* on the aggressiveness of CAC in both in vitro and in vivo colitis models using DSS, which is a well-known colitis-inducing chemical, and the related mechanism. *F. nucleatum* synergistically increased proliferation, motility, and invasion by promoting EMT in DSS-treated human CRC cells. The role of *F. nucleatum* in CAC progression was further confirmed in mouse models, as *F. nucleatum* significantly increased the malignancy of AOM/DSS-induced colon cancer. This promoting effect of *F. nucleatum* was based on activation of the EGFR signaling pathways, and EGFR inhibition significantly reduced the *F. nucleatum*-induced EMT alterations. In conclusion, *F. nucleatum* accelerates the progression of CAC by promoting EMT through the EGFR signaling pathway.

## Figures and Tables

**Table 1:** Expression profiles of CD133 and CD44 in CRC cell lines using flow cytometry.

CD133- CD44+	Control	DSS	Fn	Fn + DSS
LOVO	100 ± 1	121 ± 1	115 ± 1	117 ± 1
	100 ± 8	46 ± 2	82 ± 3	247 ± 26
HCT8	100 ± 1	116 ± 3	138 ± 6	185 ± 3
LS174T	100 ± 4	112 ± 3	138 ± 3	182 ± 13

Abbreviations: DSS, dextran sodium sulfate; Fn, *F. nucleatum*.

## Supplementary Materials

The following are available online at https://www.mdpi.com/2072-6694/12/10/2728/s1: Figure S1: The expression of *F. nucleatum* 16s rRNA in representative CRC cells detected by RT-qPCR, Figure S2: Effects of *F. nucleatum* and *P. gingivalis* on representative CRC cells, Figure S3: Observation of spleen enlargement in each mice groups, Figure S4: Representative immunohistochemical staining images for Snail and E-cadherin in colon tissue, Figure S5: Prognostic importance of epithelial to mesenchymal transition markers in patients with CRC, Figure S6: Uncropped Western Blots of Figure 2B, Figure S7: Uncropped Western Blots of Figure 6A, Figure S8: Uncropped Western Blots of Figure 7A, Figure S9: Uncropped Western Blots of Figure 7C and Figure S10: Uncropped Western Blots of Figure S2.

## References

[1] Parang B., Barrett C.W., Williams C.S. (2016). AOM/DSS Model of Colitis-Associated Cancer. Methods Mol. Biol..

[2] Jin B.R., Chung K.S., Lee M.H., An H.J. (2020). High-Fat Diet Propelled AOM/DSS-Induced Colitis-Associated Colon Cancer Alleviated by Administration of Aster glehni via STAT3 Signaling Pathway. Biology.

[3] Amitay E.L., Carr P.R., Jansen L., Roth W., Alwers E., Herpel E., Kloor M., Blaker H., Chang-Claude J., Brenner H. (2020). Smoking, alcohol consumption and colorectal cancer risk by molecular pathological subtypes and pathways. Br. J. Cancer.

[4] Lasry A., Zinger A., Ben-Neriah Y. (2016). Inflammatory networks underlying colorectal cancer. Nat. Immunol..

[5] Hnatyszyn A., Hryhorowicz S., Kaczmarek-Rys M., Lis E., Stomski R., Scott R.J., Plawski A. (2019). Colorectal carcinoma in the course of inflammatory bowel diseases. Hered Cancer Clin. Pract..

[6] Yu M., Kim J., Ahn J.H., Moon Y. (2019). Nononcogenic restoration of the intestinal barrier by E. coli-delivered human EGF. Jci Insight.

[7] Stidham R.W., Higgins P.D.R. (2018). Colorectal Cancer in Inflammatory Bowel Disease. Clin. Colon Rectal Surg..

[8] Clarke W.T., Feuerstein J.D. (2019). Colorectal cancer surveillance in inflammatory bowel disease: Practice guidelines and recent developments. World J. Gastroenterol..

[9] Yu T., Guo F., Yu Y., Sun T., Ma D., Han J., Qian Y., Kryczek I., Sun D., Nagarsheth N. (2017). Fusobacterium nucleatum Promotes Chemoresistance to Colorectal Cancer by Modulating Autophagy. Cell.

[10] Flemer B., Lynch D.B., Brown J.M., Jeffery I.B., Ryan F.J., Claesson M.J., O’Riordiain M., Shanahan F., O’Toole P.W. (2017). Tumour-associated and non-tumour-associated microbiota in colorectal cancer. Gut.

[11] Haghi F., Goli E., Mizaei B., Zeighami H. (2019). The association between fecal enterotoxigenic B. fragilis with colorectal cancer. BMC Cancer.

[12] Yan X., Liu L., Li H., Qin H., Sun Z. (2017). Clinical significance of Fusobacterium nucleatum, epithelial-mesenchymal transition, and cancer stem cell markers in stage III/IV colorectal cancer patients. Onco Targets.

[13] Yang Z.H., Ji G. (2019). Fusobacterium nucleatum-positive colorectal cancer. Oncol. Lett..

[14] Sun C.H., Li B.B., Wang B., Zhao J., Zhang X.Y., Li T.T., Li W.B., Tang D., Qiu M.J., Wang X.C. (2019). The role of Fusobacterium nucleatum in colorectal cancer: From carcinogenesis to clinical management. Chronic. Dis. Transl. Med..

[15] Liu L., Liang L., Liang H., Wang M., Lu B., Xue M., Deng J., Chen Y. (2019). Fusobacterium nucleatum Aggravates the Progression of Colitis by Regulating M1 Macrophage Polarization via AKT2 Pathway. Front. Immunol..

[16] Pastushenko I., Blanpain C. (2019). EMT Transition States during Tumor Progression and Metastasis. Trends Cell Biol..

[17] Ribatti D., Tamma R., Annese T. (2020). Epithelial-Mesenchymal Transition in Cancer: A Historical Overview. Transl. Oncol..

[18] Roche J. (2018). The Epithelial-to-Mesenchymal Transition in Cancer. Cancers.

[19] Zhou P.T., Li B., Liu F., Zhang M., Wang Q., Liu Y., Yao Y., Li D. (2017). The epithelial to mesenchymal transition (EMT) and cancer stem cells: Implication for treatment resistance in pancreatic cancer. Mol. Cancer.

[20] Ma S.Y., Park J.H., Jung H., Ha S.M., Kim Y., Park D.H., Lee D.H., Lee S., Chu I.H., Jung S.Y. (2017). Snail maintains metastatic potential, cancer stem-like properties, and chemoresistance in mesenchymal mouse breast cancer TUBO-P2J cells. Oncol. Rep..

[21] Dudas J., Ladanyi A., Ingruber J., Steinbichler T.B., Riechelmann H. (2020). Epithelial to Mesenchymal Transition: A Mechanism that Fuels Cancer Radio/Chemoresistance. Cells.

[22] Poh M.E., Liam C.K., Rajadurai P., Chai C.S. (2018). Epithelial-to-mesenchymal transition (EMT) causing acquired resistance to afatinib in a patient with epidermal growth factor receptor (EGFR)-mutant lung adenocarcinoma. J. Thorac. Dis..

[23] Xiong Y.Y., Yuan L., Chen S., Xu H., Peng T., Ju L., Wang G., Xiao Y., Wang X. (2020). WFDC2 suppresses prostate cancer metastasis by modulating EGFR signaling inactivation. Cell Death Dis..

[24] Mizumoto A., Yamamoto K., Nakayama Y., Takara K., Nakagawa T., Hirano T., Hirai M. (2015). Induction of Epithelial-Mesenchymal Transition via Activation of Epidermal Growth Factor Receptor Contributes to Sunitinib Resistance in Human Renal Cell Carcinoma Cell Lines. J. Pharmacol. Exp. Ther..

[25] Gan Y., Shi C., Inge L., Hibner M., Balducci J., Huang Y. (2010). Differential roles of ERK and Akt pathways in regulation of EGFR-mediated signaling and motility in prostate cancer cells. Oncogene.

[26] Shang F.M., Liu H.L. (2018). Fusobacterium nucleatum and colorectal cancer: A review. World J. Gastrointest. Oncol..

[27] Chen Y.Y., Chen Y., Cao P., Su W., Zhan N., Dong W. (2020). Fusobacterium nucleatum facilitates ulcerative colitis through activating IL-17F signaling to NF-kappa B via the upregulation of CARD3 expression. J. Pathol..

[28] Zhang S., Cai S., Ma Y. (2018). Association between Fusobacterium nucleatum and colorectal cancer: Progress and future directions. J. Cancer.

[29] Zhang S., Yang Y., Weng W., Guo B., Cai G., Ma Y., Cai S. (2019). Fusobacterium nucleatum promotes chemoresistance to 5-fluorouracil by upregulation of BIRC3 expression in colorectal cancer. J. Exp. Clin. Cancer Res..

[30] Ganesan K., Guo S., Fayyaz S., Zhang G., Xu B. (2019). Targeting Programmed Fusobacterium nucleatum Fap2 for Colorectal Cancer Therapy. Cancers.

[31] Pearson G.W. (2019). Control of Invasion by Epithelial-to-Mesenchymal Transition Programs during Metastasis. J. Clin. Med..

[32] Chen Y.Y., Chen Y., Zhang J., Cao P., Su W., Deng Y., Zhan N., Fu X., Huang Y., Dong W. (2020). Fusobacterium nucleatum Promotes Metastasis in Colorectal Cancer by Activating Autophagy Signaling via the Upregulation of CARD3 Expression. Theranostics.

[33] Lin L.J., Sung Y., Wang D., Zheng S., Zhang J., Zheng C. (2016). Celastrol Ameliorates Ulcerative Colitis-Related Colorectal Cancer in Mice via Suppressing Inflammatory Responses and Epithelial-Mesenchymal Transition. Front. Pharmacol..

[34] Lee J., Roberts J.S., Atanasova K.R., Chowdhury N., Han K., Yilmaz O. (2017). Human Primary Epithelial Cells Acquire an Epithelial-Mesenchymal-Transition Phenotype during Long-Term Infection by the Oral Opportunistic Pathogen, Porphyromonas gingivalis. Front. Cell Infect. Microbiol..

[35] Holt R.A., Cochrane K. (2017). Tumor Potentiating Mechanisms of Fusobacterium nucleatum, A Multifaceted Microbe. Gastroenterology.

[36] Pradella D., Naro C., Sette C., Ghigna C. (2017). EMT and stemness: Flexible processes tuned by alternative splicing in development and cancer progression. Mol. Cancer.

[37] Wang Y., Hu J., Wang Y., Ye W., Zhang X., Ju H., Xu D., Liu L., Ye D., Zhang L. (2018). EGFR activation induced Snail-dependent EMT and myc-dependent PD-L1 in human salivary adenoid cystic carcinoma cells. Cell Cycle.

