# Supplementary material for: Fusobacterium nucleatum Accelerates the Progression of Colitis-Associated Colorectal Cancer by Promoting EMT
Source: Cancers (Basel). 2020 Sep 23;12(10):2728. doi: 10.3390/cancers12102728 (PMC7598280; doi:10.3390/cancers12102728)
Supplement: Supplementary file 1 [file cancers-12-02728-s001.pdf]

Supplementary Materials: *Fusobacterium nucleatum* accelerates progression of colitis-associated colorectal cancer by promoting EMT

Mi Ra Yu, Hye Jung Kim and Hae Ryoun Park

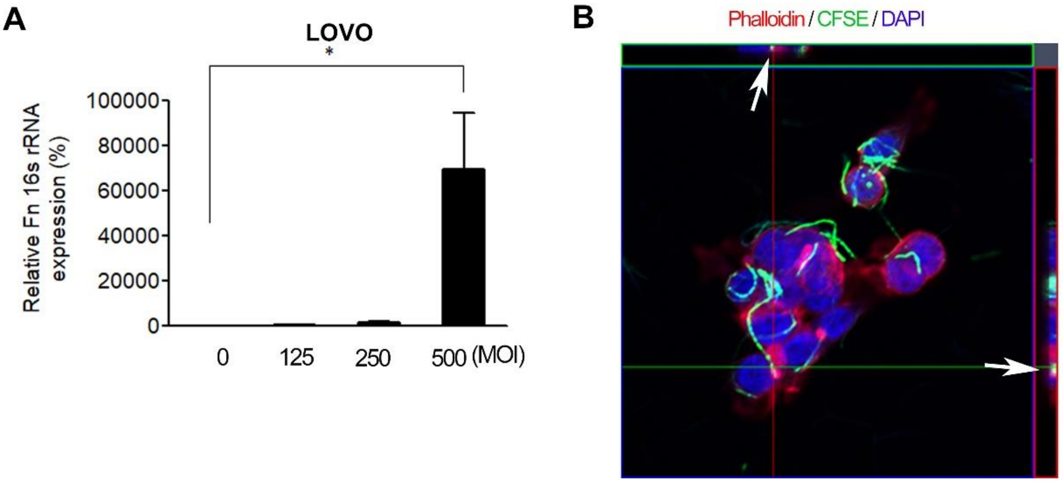

Figure 1. The expression of *F. nucleatum* 16s rRNA in representative CRC cell was detected by RT-qPCR. (**A**) Data are means ± SEM. * $p < 0.05$ vs. corresponding control. (**B**) Orthogonal view from different planes (x/y, x/z or y/z) of the confocal microscope images used to confirm internalization of *F. nucleatum* within CRC cells. Filled arrows indicate internalized in cytoplasm.

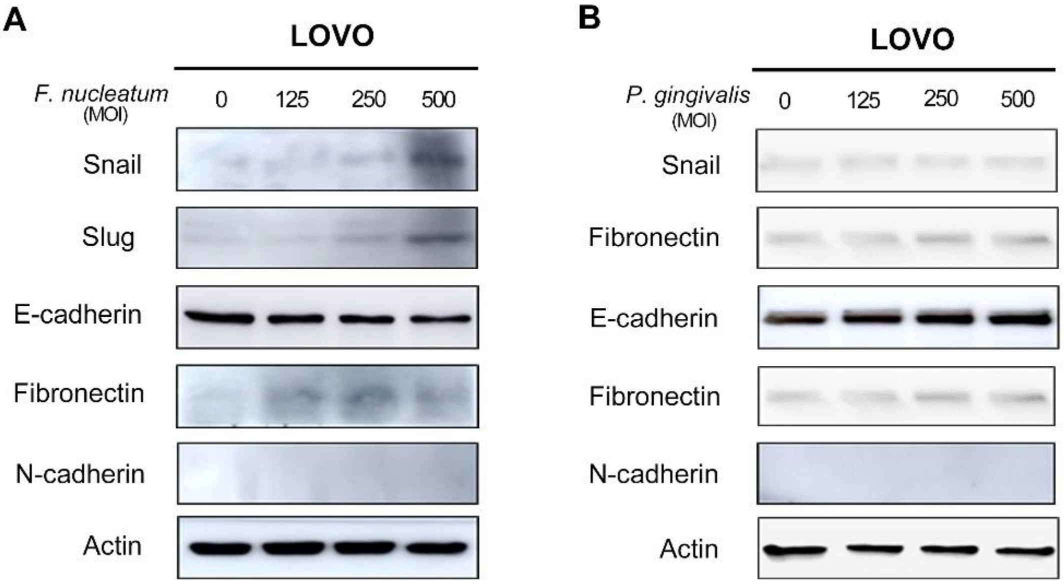

Figure 2. Effect of *F. nucleatum* and *P. gingivalis* in representative CRC cell. Expression of EMT markers in (**A**) *F. nucleatum* and (**B**) *P. gingivalis* infected CRC cell was detected by western blot analysis. Uncropped Western Blots can be observed in Figure S10.

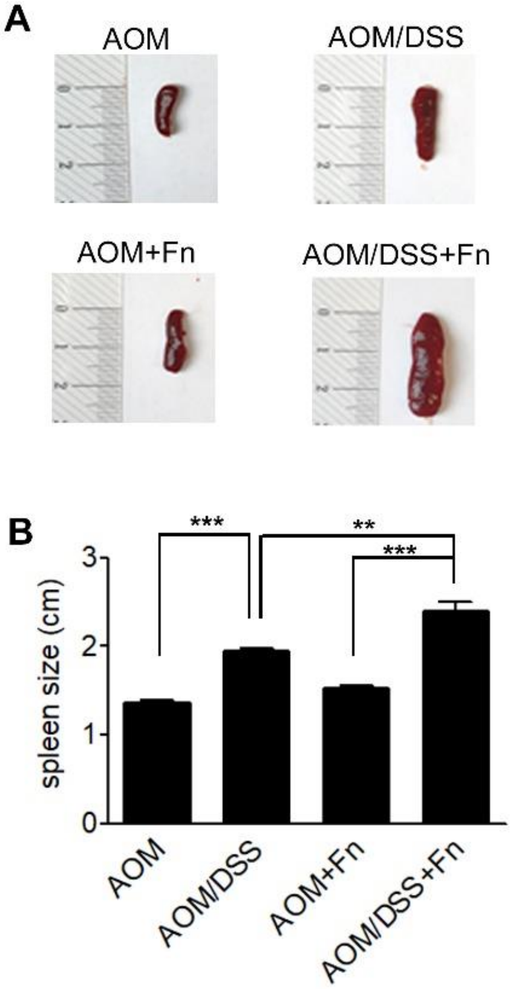

Figure 3. Observation of spleen enlargement in each mice groups. (**A**) Gross images of spleen. (**B**) graphical quantitation. Data are presented as the means ± SEM (**p < 0.01, and ***p < 0.005; unpaired Student's t-test).

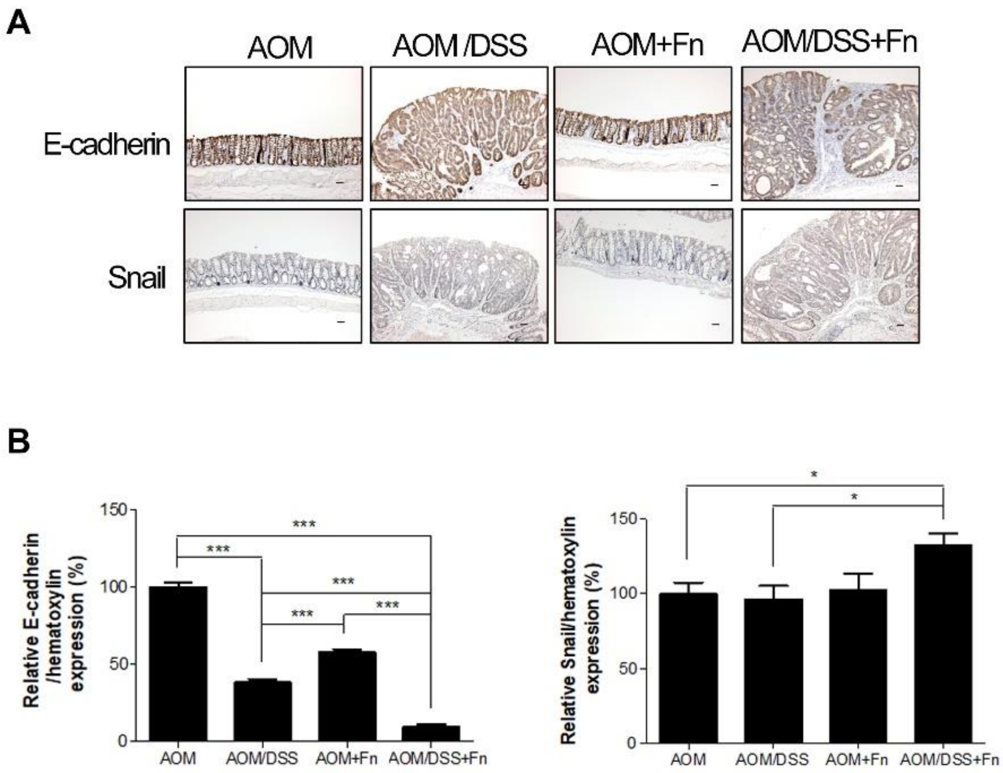

Figure 4. Representative immunohistochemical staining images for Snail and E-cadherin in colon tissue. (**A**) Microscopy image at the original magnification of 200 times is shown. (**B**) The graph represents relative protein expression levels obtained from immunohistochemical staining images for E-cadherin and Snail compared with the AOM group. The data are presented as the mean ± SEM (**p* < 0.05 and ****p* < 0.001; unpaired Student's *t*-test). Abbreviations: AOM, azoxymethane; DSS, dextran sodium sulfate; Fn, *F. nucleatum*.

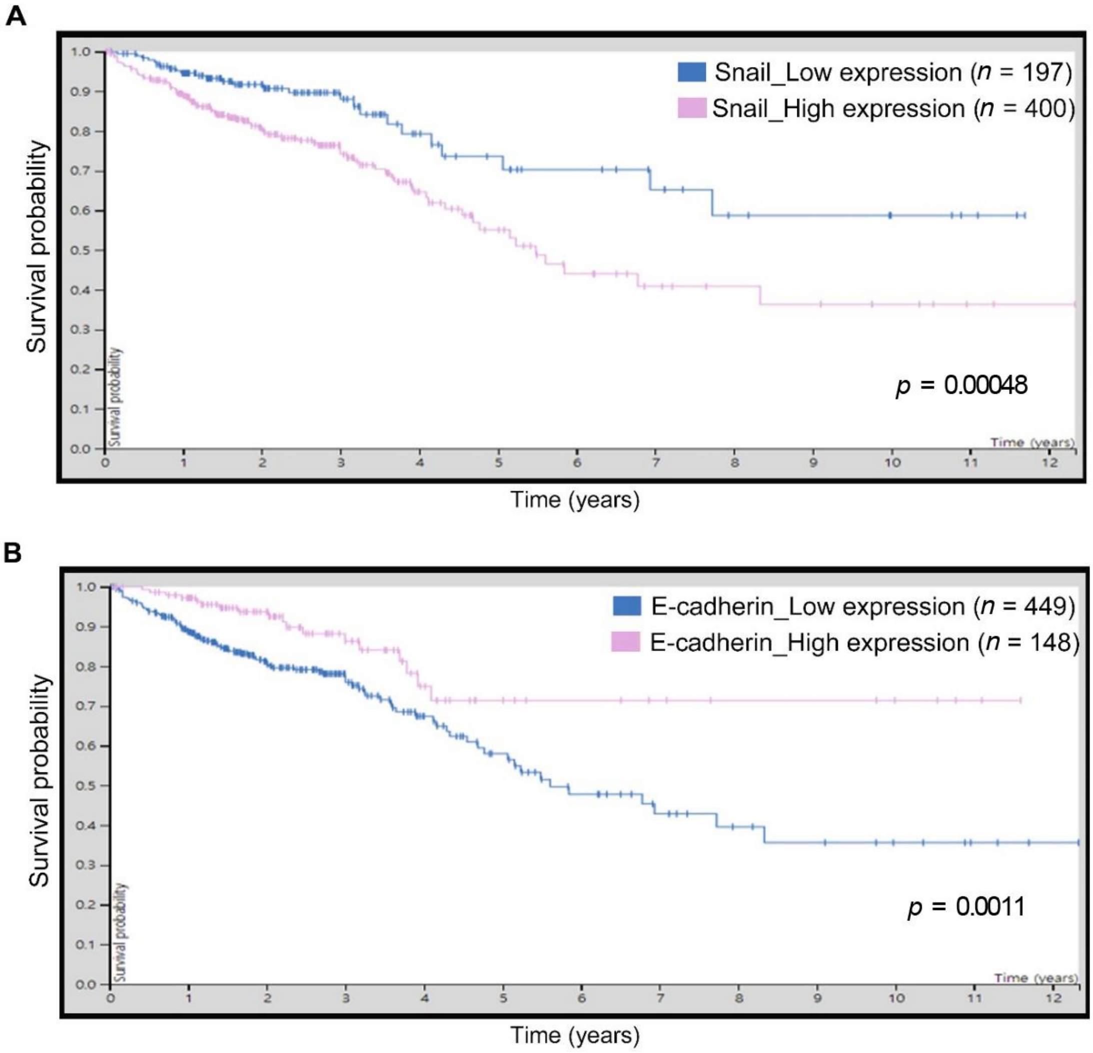

Figure 5. Prognostic importance of epithelial to mesenchymal transition markers in CRC patients. (**A**) Survival probability curves stratified by Snail expression. (**B**) Survival probability curves stratified by E-cadherin.

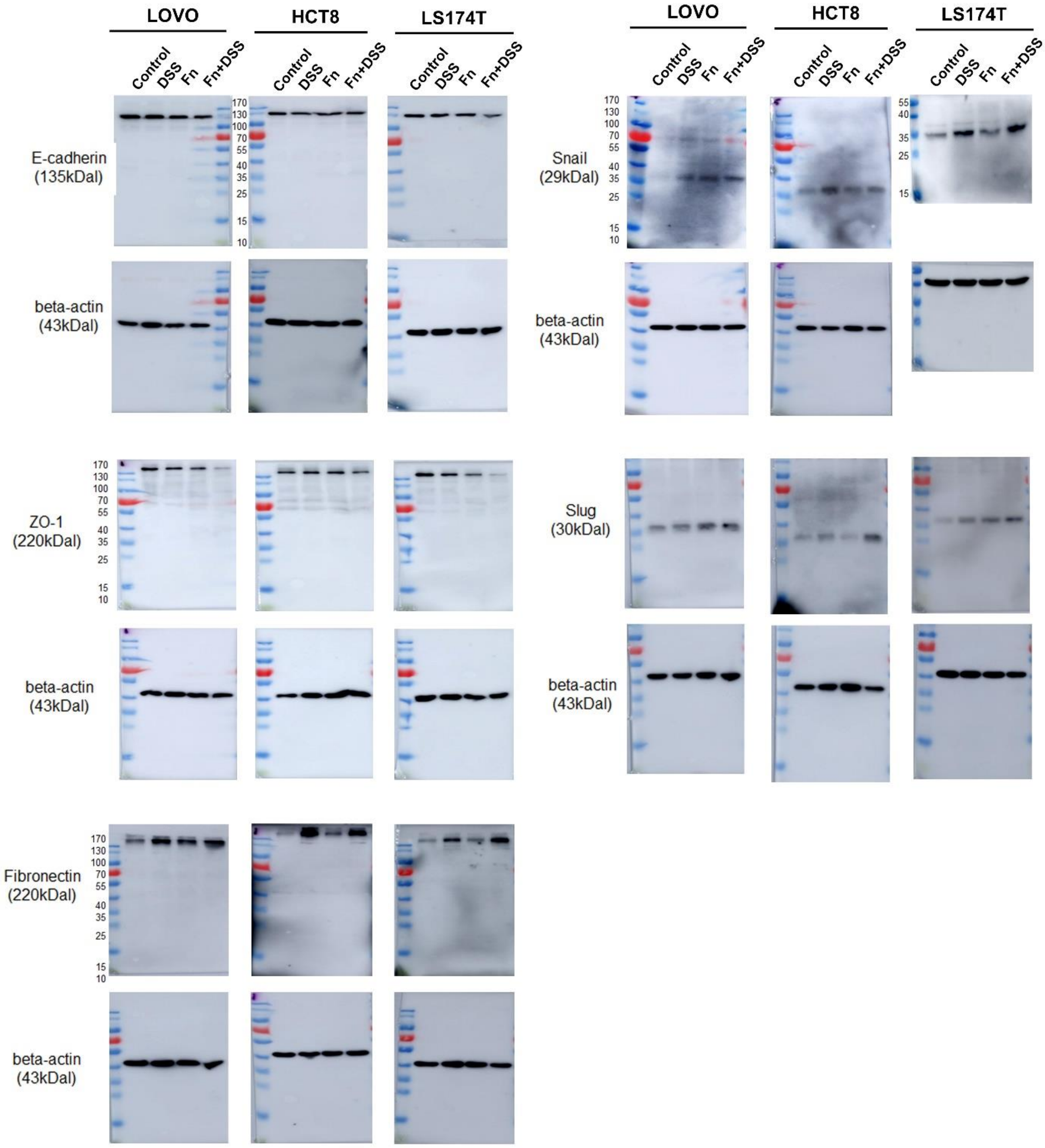

Figure 6. Uncropped Western Blots of Figure 2B.

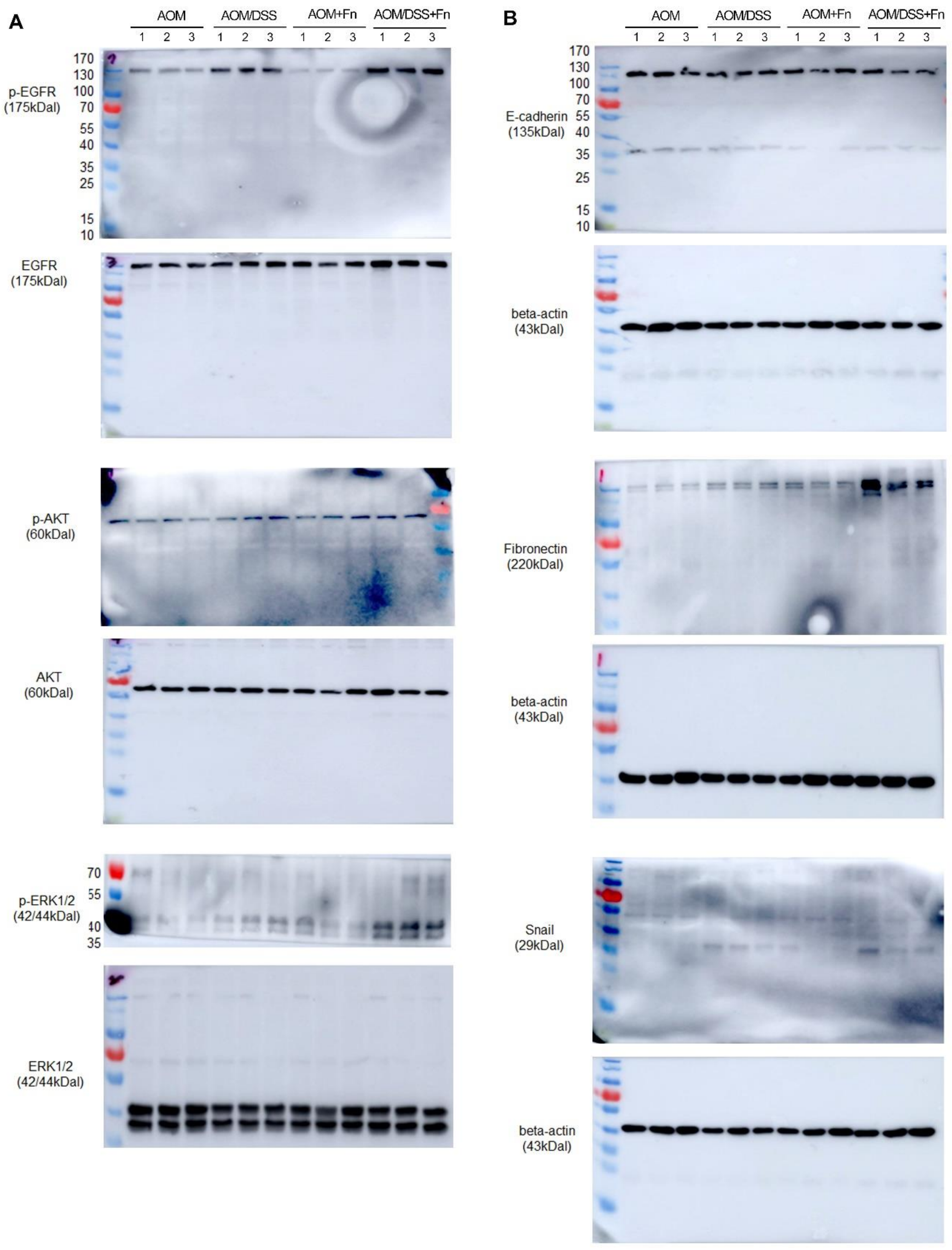

Figure 7. Uncropped Western Blots of Figure 6A.

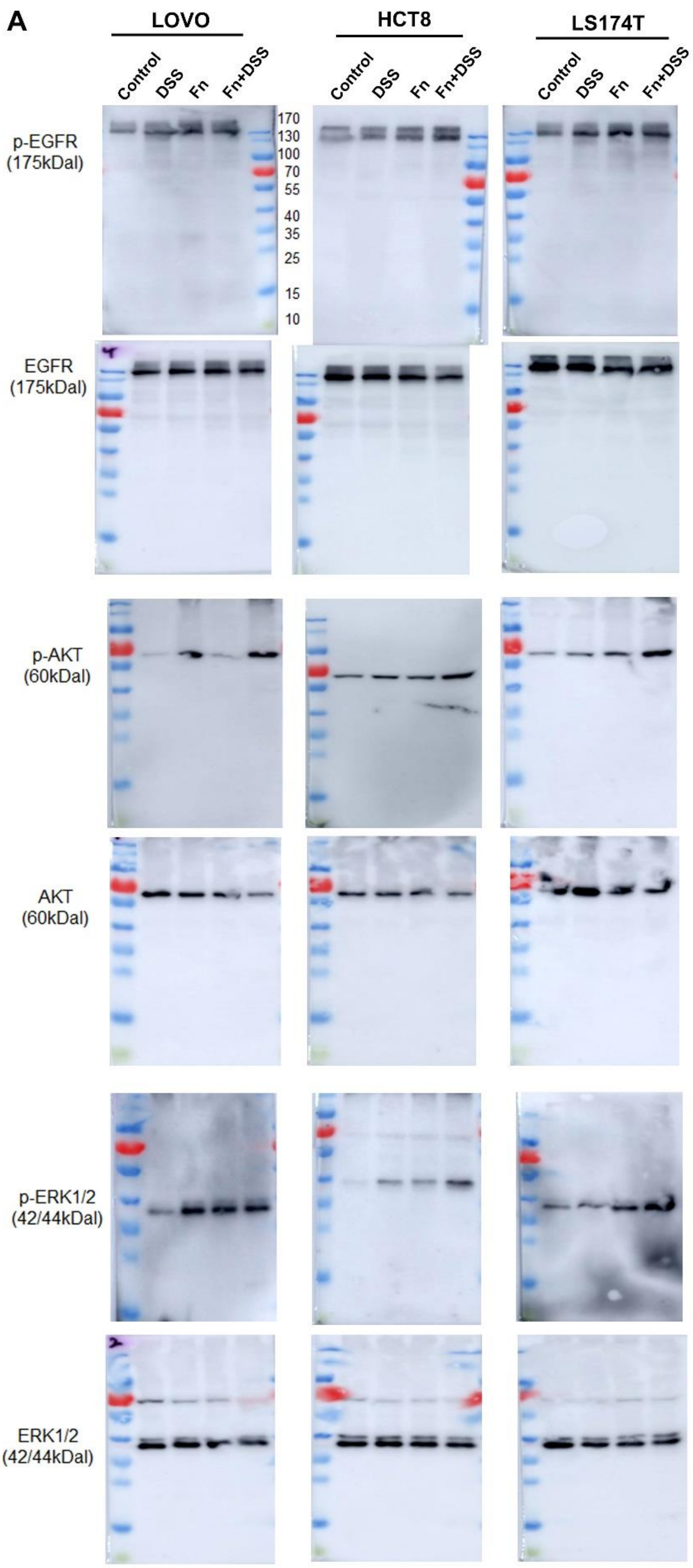

Figure 8. Uncropped Western Blots of Figure 7A.

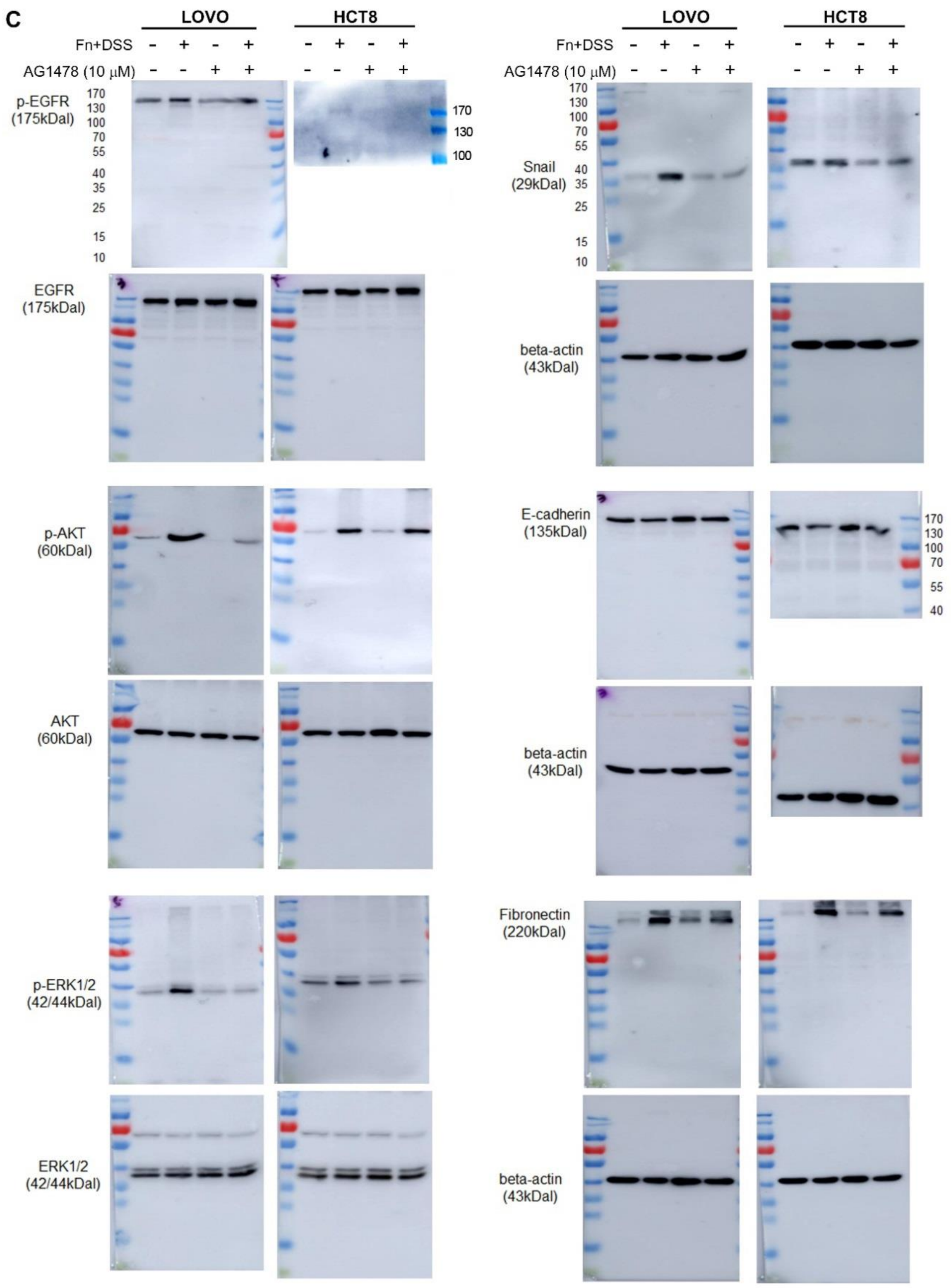

Figure 9. Uncropped Western Blots of Figure 7C.

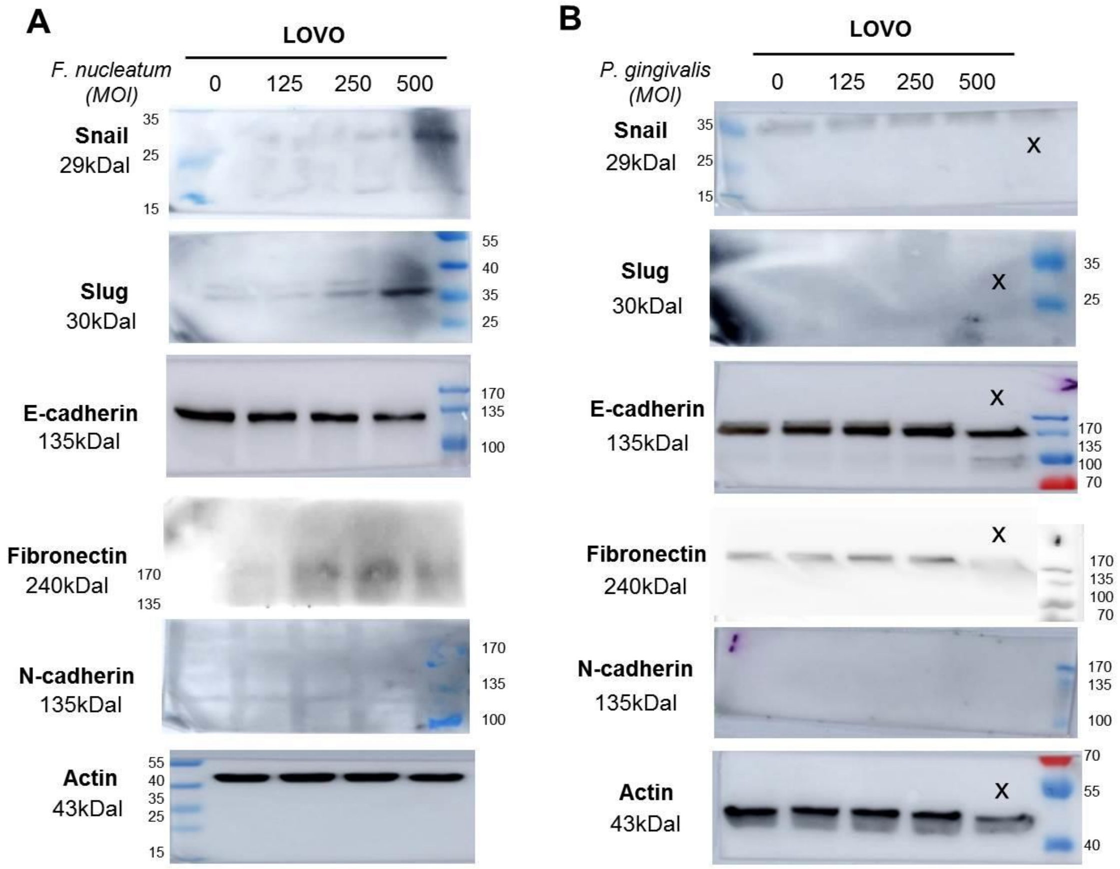

Figure 10. Uncropped Western Blots of Supplementary Material Figure 2. In (**B**), **X** indicates that it was excluded from the results because cytotoxicity was exhibited by treatment with a high concentration of *P. gingivalis*.

© 2020 by the authors. Licensee MDPI, Basel, Switzerland. This article is an open access article distributed under the terms and conditions of the Creative Commons Attribution (CC BY) license (http://creativecommons.org/licenses/by/4.0/).
